# PReS-FINAL-2160: Intestinal microbiome in polyarticular juvenile idiopathic arthritis: a pilot study

**DOI:** 10.1186/1546-0096-11-S2-P172

**Published:** 2013-12-05

**Authors:** PC Hissink Muller, PM Westedt, AE Budding, CF Allaart, DM Brinkman, TW Kuijpers, JM Van den Berg, LW Van Suijlekom-Smit, MA Van Rossum, TG De Meij, R Ten Cate

**Affiliations:** 1 Pediatric Rheumatology, Reade, location Jan van Breemen, Amsterdam, Netherlands; 2 Pediatric Rheumatology, LUMC, Leiden, Netherlands; 3 Medical Microbiology, VU Medical Center, Amsterdam, Netherlands; 4 Rheumatology, LUMC, Leiden, Netherlands; 5 Pediatrics, Rijnland Hospital, Leiderdorp, Netherlands; 6 Pediatric Rheumatology, Academic Medical Center, Emma Children's Hospital, Amsterdam, Netherlands; 7 Pediatric Rheumatology, Erasmus MC, Sophia Children's Hospital, Rotterdam, Netherlands; 8 Pediatric Rheumatology, Reade location Jan van Breemen, Netherlands; 9 Pediatric Gastroenterology, VU Medical Center, Amsterdam, Netherlands

## Introduction

The intestinal microbiome may play a role in the pathogenesis of Juvenile Idiopathic Arthritis (JIA). In IBD patients an overall decrease in microbial diversity of the intestinal microbiota has been observed. Studies comparing intestinal microbiome in children with JIA and healthy controls have not been conducted to date.

## Objectives

To analyse and compare the composition and diversity of the distal colon associated microbiome between children with Disease-Modyfying-Anti-Rheumatic-Drug (DMARD) naive JIA and healthy controls and to identify specific gut bacteria associated with JIA before initiation of a DMARD.

## Methods

Total microbiome profile in stools of 8 children with DMARD naive polyarticular JIA were analyzed by means of IS-pro, a 16S-23S interspacer (IS) region-based profiling method and compared to stools of 24 age-matched healthy controls.

## Results

Faeces of 8 (6 girls, 2 boys) children with polyarticular JIA, all rheumatoid factor negative were investigated and compared to 24 healthy controls. Anti-Nuclear Antibodies were positive in 3 patients. Median age at evaluation was 11.1 years (7.3-13.1), median period from start complaints to diagnosis was 7.1 months (4.4-13.2). Median ACR pedi scores were: VAS physician 47 mm(32-58), VAS patient well-being 32 mm(27-52), ESR 8 mm(2-9), active joint count 10(7-14), limited joint count 2 (0-4), CHAQ score 1.2 (0.4-1.7).

One intra-articular steroid injection was given to each of two patients respectively 1 and 4 months prior to stool collection. Non Steroidal antiinflammatory Drugs (nsaids) were used by all patients at the time of evaluation. Median age of the healthy controls was 10.6 years (8.4-12.9).

The median Simpsons' diversity index within the phylum Firmicutes in controls and JIA was 0,88, and 0,83 respectively (p < 0,012). Diversity within the phyla Bacteriodetes and Proteobacteria did not differ between the 2 subgroups. By constructing a Pearson-correlation dendogram, no clustering was seen between the JIA group and the healthy controls on species-level (figure 2), a specific JIA associated microbiotal signature could not be identified.

## Conclusion

Intestinal microbiome diversity within the phylum Firmicutes was significantly lower between children with DMARD naive polyarticular JIA and healthy controls. An overall decrease in microbial diversity of the intestinal microbiota has also been observed in IBD patients. Whether intestinal dysbiosis plays a role in the pathogenesis of JIA remains subject of further studies.

## Disclosure of interest

None declared.

